# Process Simulation and Environmental Aspects of Dimethyl Ether Production from Digestate-Derived Syngas

**DOI:** 10.3390/ijerph18020807

**Published:** 2021-01-19

**Authors:** Aristide Giuliano, Enrico Catizzone, Cesare Freda

**Affiliations:** ENEA–Italian Agency for New Technologies, Energy and Sustainable Economic Development, Energy Technologies and Renewable Sources Department, Trisaia Research Centre, I-75026 Rotondella, Italy; aristide.giuliano@enea.it (A.G.); enrico.catizzone@enea.it (E.C.)

**Keywords:** dimethyl ether, digestate, gasification, process simulation, carbon footprint

## Abstract

The production of dimethyl ether from renewables or waste is a promising strategy to push towards a sustainable energy transition of alternative eco-friendly diesel fuel. In this work, we simulate the synthesis of dimethyl ether from a syngas (a mixture of CO, CO_2_ and H_2_) produced from gasification of digestate. In particular, a thermodynamic analysis was performed to individuate the best process conditions and syngas conditioning processes to maximize yield to dimethyl etehr (DME). Process simulation was carried out by ChemCAD software, and it was particularly focused on the effect of process conditions of both water gas shift and CO_2_ absorption by Selexol^®^ on the syngas composition, with a direct influence on DME productivity. The final best flowsheet and the best process conditions were evaluated in terms of CO_2_ equivalent emissions. Results show direct DME synthesis global yield was higher without the WGS section and with a carbon capture equal to 85%. The final environmental impact was found equal to −113 kgCO_2_/GJ, demonstrating that DME synthesis from digestate may be considered as a suitable strategy for carbon dioxide recycling.

## 1. Introduction

Carbon dioxide is recognized as the main greenhouse gas (GHG), causing dramatic changes in the Earth’s climate. In this concern, in order to avoid more dangerous climate change effects, the Intergovernmental Panel on Climate Change (IPCC) and the United Nations Climate Change Conference (COP21, Paris, 2015) have emphasized the necessity to reduce carbon dioxide emissions by at least one half of the current trend until 2050, aiming to limit the global average temperature increases to a maximum of 2 °C [1]. Transportation, industry and power plants are the main sources of CO_2_ emissions, which are expected to increase in the next years due to the growth of the energy demand. Therefore, in the last decades, several strategies and technologies have been developed to capture and store carbon dioxide (CCS systems). During the last years, growing attention has been devoted to the reuse of carbon dioxide as carbon source (C-source) for the production of chemicals and fuels in a sustainable way [2]. Besides CO_2_ emission issues, waste management is another big point to be addressed in order to improve and preserve both public and environmental health. In particular, the management of municipal solid waste (MSW) represents a very challenging issue in our society. In fact, the municipal solid waste generation is expected to increase from 3.5 Mtons/day in 2010 to more than 6 Mtons/day in 2025. MSW is a heterogeneous stream which consists of several kinds of waste, such as paper, plastics, textile, glass, food residues and so on [3]. A well-organized differentiated collection of waste is the first step for efficient management and disposal of waste. Differentiate collections indeed allows to separate the different fractions, which may be recycled or valorized. The composition of MSW significantly depends on several factors, such as lifestyle, society welfare, geopolitical framework and knowledge on the management of waste [4]. The organic fraction (OFMSW), which mainly consists of food residues of both domestic and industrial origin, represents one of the main fraction of MSW, with about 46% worldwide waste generation. It is forecasted that the generation of OFMSW will gradually grow over the coming years, reaching more than 11 Mtons per day in 2100, which is more the three times the current generation rate. Therefore, disposal in landfill sites cannot be considered the most sustainable solution. In fact, due to ever more stringent environmental regulations besides the increasing demand for renewable fuels, the management of OFMSW is ever more devoted to considering OFMSW as C-source for the production of alternative fuels [5]. In this sense, the most commonly used technology for the recycling of OFMSW is composting and anaerobic digestion. Composting is an aerobic biological method to decompose the organic fraction into humus-like materials, known as compost, which may be used as a fertilizer for plants. On the contrary, anaerobic digestion is a biological method to convert organic fractions into biogas and digestate. Biogas, a mixture of methane and carbon dioxide, is usually directly used as an energy source for on-site combined heat and power generation. During the last years, growing attention has focused on upgrading biogas to produce bio-methane, to be used as an alternative to natural gas, in either compressed or liquefied form, for the transport sector [6]. Due to bio-methane production’s profitability, the number of anaerobic digestion plants is expected to increase in the next years. The other stream produced from anaerobic digestion process, namely digestate, is usually sent to a composting step to produce compost. Both the low cost and the high production rate of digestate push towards a more profitable valorization of this stream [7,8]. In particular, digestate may be considered a C-source that may be exploited to produce chemicals and fuels. In this regard, gasification of digestate has been explored as a technology to convert digestate into syngas, a mixture of CO, CO_2_ and H_2_, which may be further converted into high added value products [9]. 

Gasification is partial oxidation of an organic feedstock that occurs in a temperature range of 700–900 °C. The most common oxidant agents are air, oxygen, steam or their mixture. They are fed to the gasifier in a substoichiometric amount compared to the quantitative combustion of the feedstock. The equivalence ratio in the range 0.2–0.4 is usually used. The equivalence ratio is defined as the ratio between the amount of oxidant fed to the gasifier and the amount of oxidant required for the quantitative combustion. The organic feedstock that undergoes to gasification usually has a moisture content in the range 5–15%wt. Higher moisture content severely can affect the cold gas efficiency of the gasification. This latter is defined as “(thermal power of the syngas)/(thermal power of feedstock and input thermal power)”. Gasification can be conducted in autothermal mode when a proper equivalence ratio is set. In the autothermal mode, the heat to sustain the endothermic gasification reactions is provided by the exothermic reactions, e.g., oxidation reactions. Usually, industrial gasification plants work in autothermal mode, and several productive experiences have been acquired worldwide in last few decades. The main product of the process is syngas, whose composition is related to the feedstock composition and oxidant agent. Compared to the feedstock, syngas is more versatile; in fact, it can be burnt, after proper cleaning, in internal combustion engines, gas turbines and fuel cells [10,11]. The interest and development towards the gasification have been inversely linked to the availability of alternative technology and fuels. Historically, in the 1800s coal gasification was developed to produce town gas for lighting and cooking before the advent of large-scale production of natural gas from oil wells. During the Second World War in Europe, because of the rationing of fossil fuels, trucks, buses, tractors, motorcycles, ships, boats and trains were equipped with gasifiers to produce gas for traction. With the oil crisis of 1973 a renewed interest in the wood gasification occurred. From a technical point of view, the organic contaminants of the syngas, the so-called tar, are the main current obstacle to overcame [12]. In past years, the industrial government’s policies have been strongly driven by environmental issues such as greenhouse gas emissions and waste management. This concern for the environment lays the foundations for ongoing research in the field of gasification of waste or waste-derived streams, especially if it is addressed towards the production of green chemicals (e.g., DME, methanol). As mentioned before, digestate is a stream derived from municipal solid waste management, which needs to be further valorized, with respect to the actual utilization as feedstock for compost production. Few research works are available in the literature concerning gasification of digestate, although it may be considered a strategy to produce syngas in a sustainable way [13,14,15].

In fact, syngas is currently produced from fossils, such as coal, natural gas and crude oil fractions, by consolidated technologies. Steam reforming, partial oxidation and autothermal reforming are the most adopted process to produce syngas. Syngas gas is usually used for the production of hydrogen or methanol. Hydrogen is largely used in conventional refinery, bio-refinery and ammonia synthesis plants, while methanol is one of the most important intermediates of the industrial chemistry. The development of technologies for the production of hydrogen from renewables, such as the electrolysis of water fueled by solar energy, will reduce hydrogen production from fossils in the future [16]. On the contrary, a C-source will need to produce methanol and its derivatives, such as formaldehyde, bio-diesel, olefins, aromatics and dimethyl ether. Therefore, gasification of biomass or waste is a strategy to produce syngas within a concept of the circular and low carbon economy. In this regard, the conversion of biomass/waste-derived syngas to dimethyl ether (DME) is receiving growing attention from scientific research, as DME is a candidate to become a valuable energetic vector of the future. 

DME, the simplest of the ethers with chemical formula C_2_H_6_O, is neither a toxic nor carcinogenic molecule, which is currently used as an aerosol propellant and alternative LPG fuel. The suitability of DME as an alternative fuel for diesel engines is well-recognized, thanks to its cleaner emissions with respect to conventional diesel fuel, in terms of SOx, NOx and particulate matter [17]. Furthermore, DME is also considered as a substitute to methanol (MeOH) for both olefins and synthetic-gasoline production [18,19,20,21].

DME is produced from syngas following either an indirect route or direct route.

The indirect route, the traditional way to produce DME, is a double-step process. In the first step methanol is synthesized from syngas over Cu/ZnO-based catalysts at 240–280 °C and 3–7 MPa, followed by the second step which is the dehydration of the methanol by the following reaction:

2CH_3_OH = CH_3_OCH_3_ + H_2_O (1)

Methanol dehydration is an exothermic reversible reaction (−∆H°298 K = 23.5 kJ/mol) that proceeds without mole number variation. For this reason, operation pressure does not affect equilibrium conversion, while low reaction temperatures have a thermodynamic benefit toward DME production. Methanol dehydration is a reaction catalyzed by acid catalysts, and several investigations have been published in order to individuate an active, selective and stable catalyst at relatively low temperatures for the above-mentioned thermodynamic advantages. Depending on catalyst characteristics, methanol dehydration can be carried out in both vapor and liquid phases, with reaction temperatures in the range 100–300 °C and pressure up to 20 bar. γ-Al_2_O_3_ is the traditional catalyst for vapor phase methanol dehydration [22]. In the direct route, both the synthesis and the consecutive dehydration of methanol are carried out in a single reactor over a redox/acid bi-functional catalyst under process conditions close to those of the methanol synthesis, and the following reactions are involved:

CO + 2H_2_ = CH_3_OH
CO_2_ + 3 H_2_= CH_3_OH + H_2_O
CO + H_2_O = CO_2_ + H_2_
2CH_3_OH = CH_3_OCH_3_ + H_2_O (2)

The main advantage of the direct synthesis route to DME is surpassing the equilibrium constrain of the methanol synthesis step by the in situ removal of methanol, resulting in a higher CO/CO_2_ conversion.

Several works were devoted to the assessment of the synthesis of DME from biomass or waste from both environmental and economic points of view, with the aim to give insights about the viability and sustainability of the proposed strategy. 

Ju et al. [23] simulated the one-pot process of DME synthesis via woody biomass gasification. The process scheme proposed by the authors comprises an oxygen/steam gasifier simulated for different steam-to-oxygen ratios. Due to the low H_2_/CO ratio in the producer syngas, the authors proposed an upgrading section which consists of a water-gas-shift (WGS) unit followed by a purification system. The WGS serves to increase the H_2_/CO ratio up to 1 as in the JFE technology, although a ratio equal to 2 may also be adopted, as in the Haldor–Topsoe process. With the purification unit, the authors simulated the removal of H_2_S and CO_2_ produced from gasification. In particular, a maximum of 3% of CO_2_ in the syngas was allowed. The authors also simulated the production of electricity from a surplus of energy. Globally, the DME yield was 0.37, assuming a 0.91 DME selectivity and a unitary conversion of CO. With respect to biomass combustion, the production of DME reduced CO_2_ emissions by 26.7%.

Clausen et al. [24] recently published a study on the production of DME from terrified wood biomass with the aim to compare the following two cases: (i) recycle of unreacted syngas to DME reactor in order to maximize the DME productivity (RC plant), and (ii) utilization of unreacted syngas for electricity production in a combined cycle (OT plant). After gasification of the terrified biomass in an oxygen/steam gasifier, the produced syngas was sent to a WGS section to increase the H_2_/CO up to 1 or 1.6, in the case of RC or OT plant, respectively. For this purpose, a carbon capture storage stage was also simulated. The calculated CO_2_ emissions with respect to the biomass feed were 22% and 28% for RC and OT process schemes, respectively. In that case, the maximum CO_2_ level in the DME reactor inlet was set to 3%. 

The H_2_/CO ratio is of paramount importance for DME synthesis. Lu et al. indicated an H_2_/CO = 1 as optimum value for one-pot DME synthesis using a fluidized bed reactor, as also reported by Ogawa et al. [25] and Huang et al. [26]. On the contrary, an H_2_/CO ratio of 2 was indicated as a value suitable for obtaining a high DME yield by Moradi et al. [27] and Peng et al. [28]. Therefore, the proper H_2_/CO ratio for DME synthesis is the range 1–2, as a function of the process scheme [29]. 

Recently, environmental and climate change have been emphasized, and the recycling of carbon dioxide as a substitute to CO for DME synthesis has received growing attention. In particular, the hydrogenation of CO_2_ to methanol or DME may be considered a key strategy to introduce renewables in the chemical industry chain, in the case of hydrogen produced from renewable sources [30,31]. Nevertheless, DME yield is negatively influenced by the CO_2_ content in the reactor feedstock. In fact, in the case of CO_2_-rich feeds, reverse water gas shift is promoted, causing water accumulation, which limits both effective water removal by WGS and methanol dehydration step. Furthermore, the presence of a higher amount of water promotes a faster metal sintering and metal oxidation of the catalytic phase, causing catalyst deactivation. Therefore, the in situ water removal or the development of a water-resistant catalyst would be useful to bring benefits to the process [32,33]. The production of DME for CO_2_-rich syngas should also be simulated, especially for assessing the possibility of using CO_2_ as a partial or total substitute of CO. 

In this work, a novel methodology to individuate and to assess the techno-environmental performances of the optimal flowsheet of the digestate valorization by DME production was approached. Using computer-aided process simulation with ChemCAD software, the production of dimethyl ether via direct synthesis from a syngas derived from gasification of digestate was modelled on three different levels in series. After a thermodynamic analysis of the single-pass DME synthesis, the work focuses on the process simulation of DME synthesis by conversion of either produced CO_2_-rich raw syngas considering the syngas recycle. In particular, WGS conversion, CO_2_ capture and purge ratio were varied to find the optimal process configuration. Finally, the best flowsheet and process conditions were set to evaluate the bio-DME’s environmental impact analysis derived by digestate gasification.

## 2. Materials and Methods 

This work was carried out by three steps of process analysis:

(1) Thermodynamic analysis to know how the syngas composition, temperature and pressure had an impact on thermodynamic equilibrium;
(2) Thermodynamic and process analysis to individuate the best combination of process parameters (detailed list is shown in Table 1) maximizing the total yield to DME;
(3) Process simulation and evaluation of main process streams and utility consumptions;
(4) Environmental impact analysis of bio-DME.

**Figure 1:**
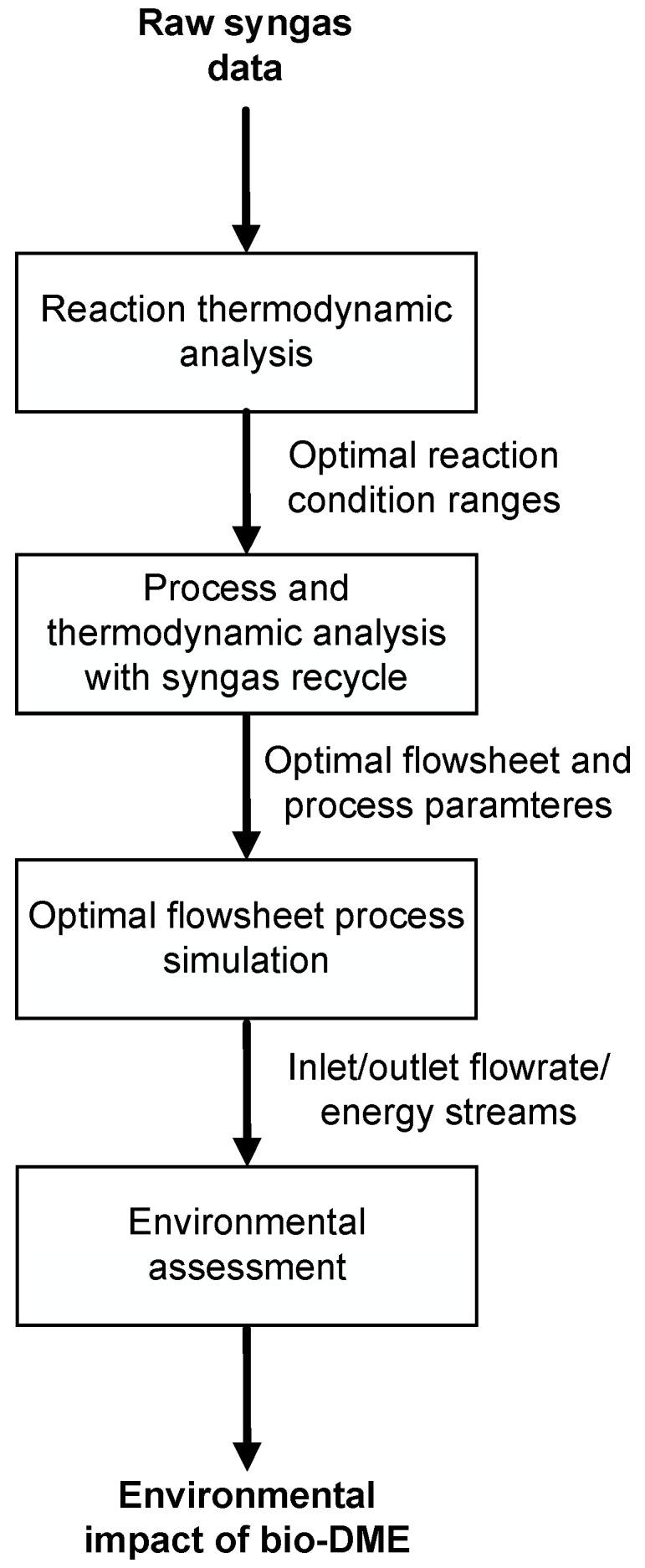
Procedure scheme to obtain the optimal bio-DME environmental assessment.

Figure 1 shows the procedure to identify the optimal flowsheet to be evaluated through the final environmental impact analysis. The scheme is based on the first thermodynamic step to know the process conditions and composition ranges to optimize the reaction yield to DME. The second step consists of the simulation of the equilibrium reactor, considering WGS, CO_2_ capture and the syngas recycling, setting the optimal process parameters of the previous step. The next step is the detailed simulation of whole DME process production, evaluating energy consumptions and material streams in input and output to assess the next environmental analysis based on the equivalent CO_2_ emissions calculation.

**Figure 2:**
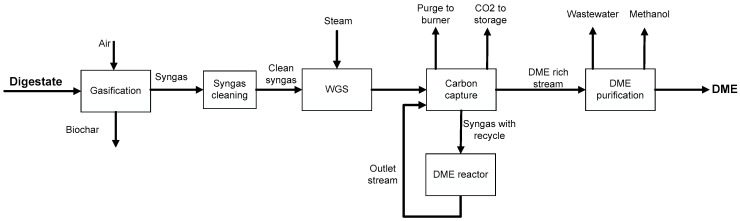
Block flow diagram of the production of DME by gasification of digestate and direct synthesis.

Figure 2 shows the block flow diagram of DME production by direct synthesis using syngas from digestate gasification.

Digestate with about 10%wt of moisture is gasified by air in a rotary kiln, which operates in autothermal mode to produce syngas and biochar. The clean syngas can be sent to the WGS section to convert carbon monoxide into CO_2_ and hydrogen, changing H_2_/CO and CO_2_/CO ratios. A reactant is the low-pressure steam. In the carbon capture section, fresh syngas and recycling were mixed to send this mix to the Selexol^®^ process and carbon dioxide storage changing the CO_2_/CO ratio. Syngas with recycle was sent to the direct DME synthesis reactor. Outlet stream was sent to the separation of volatile compounds and DME absorption by water. After that, the DME-rich stream was sent to a distillation column sequence to obtain pure DME, wastewater and methanol.

### 2.1. Thermodynamic Analysis of Direct DME Synthesis from CO_2_-Rich Syngas

The thermodynamic analysis was carried out by thermodynamic model Soave Redlich-Kwong, setting as syngas the composition of Freda et al. [15] without inert compounds (N_2_, CH_4_). Only the equilibrium reactor was set in the simulation, and CO_2_ flowrate, equilibrium temperature and pressure were varied. In particular, equilibrium temperature and pressure were varied in the range shown in Table 1. The highest pressure was 80 bar, and this is the typical pressure of the methanol synthesis [34]. The lowest pressure was 20 bar because in this analysis only CO, H_2_ and CO_2_ were considered. In the syngas derived by digestate, partial pressures of CO, H_2_ and CO_2_, consequently, can decrease until 5–10 bar. With a fixed CO flowrate, in the first case the H_2_ flowrate followed the same H_2_/CO ratio as that of the raw syngas of Freda et al. [15], 0.87; in the second case, hydrogen was set to have H_2_/CO equal to 2. In order to vary the CO_2_/CO ratio, for both cases the CO_2_ flowrate was varied to obtain CO_2_/CO between 0 and 3.

### 2.2. Thermodynamic and Process Analysis of Direct DME Synthesis from CO_2_-Rich Syngas

**Figure 3:**
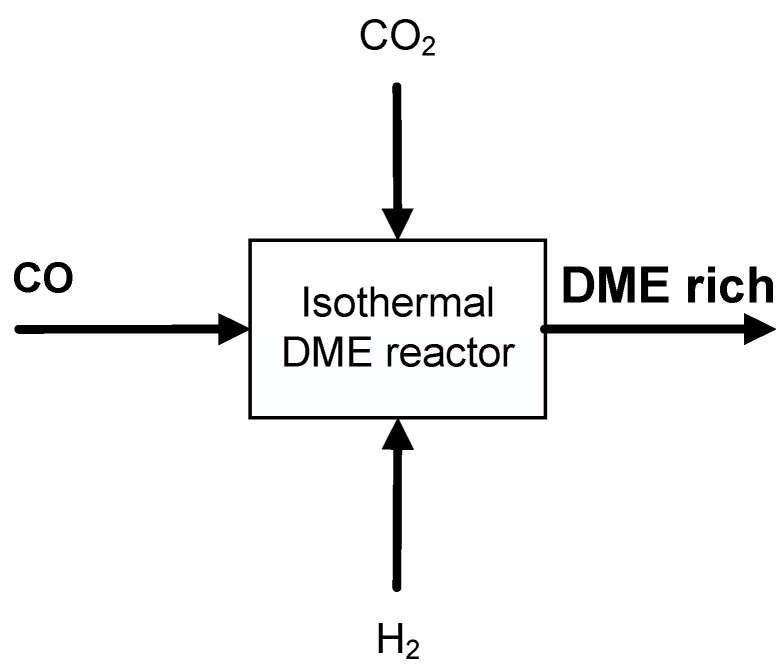
Thermodynamic analysis scheme.

In the simplified block flow diagram of Figure 3, several process parameters can be modified to obtain different global DME yields. The DME production depends on reaction conditions studied in the previous paragraph and on recycle stream contents of CO_2_, CO, H_2_ and inerts.

To have low CO_2_/CO ratios, the carbon capture has to be carried out after mixing fresh syngas and recycling. In recycling the CO_2_ fraction, the CO_2_ fraction in the fresh syngas is higher because the WGS reaction happens in the DME synthesis reactor. When DME synthesis happens, water is produced. The water can react with CO to obtain H_2_ and CO_2_ by WGS. In a process flowsheet, recycling the CO_2_ can increase its molar fraction. Consequently, CO_2_ capture has to happen after mixing in order to obtain the best CO_2_/CO ratio. In particular, a sensitivity analysis was carried out on WGS conversion, carbon capture percentage and the purge ratio in the ranges shown in Table 1.

### 2.3. Process Simulation of the Optimal Flowsheet

After the process analysis to individuate the optimal flowsheet and process conditions, a detailed process simulation was performed. Process design and optimization techniques applied to biomass valorization processes were used [38,39]. In particular, process simulation provided pumps, compressors, equilibrium reactors, distillation columns, flash, valves, absorption columns and engines. The heat integration was applied considering energy fluxes of each heat exchanger of the flowsheet.

WGS was considered to increase the partial pressure of CO_2_ and to increase the H_2_/C ratio. A double-step HT-WGS/LT-WGS was performed, and the H_2_O/CO ratio was varied between 1 and 3 to set the WGS conversion [40]. The degree of freedom of the Selexol^®^ process consisted of the syngas pressure and the solvent flowrate [41]. The DEGP solvent was varied to obtain the optimal CO_2_ capture percentage. Optimal inlet temperature and pressure of the DME synthesis reactor were set, and an adiabatic reactor was used to simulate better real industrial DME reactors. Purge gas was sent to a burner/compressor/turbine system in order to simulate the electricity production by engines [42].

### 2.4. Environmental Assessment

The environmental assessment was carried out using the simplified approach based on the equivalent CO_2_ emissions of the plant [43].

Table 2 shows the equivalent CO_2_ indices for the main process streams. The pure methanol stream was evaluated as a substitute of the fossil-based methanol. Savings due to digestate utilization will lessen the environmental impacts from transportation and avoid storage in the landfill [44]. Process water was calculated from the sum of steam to WGS and water for the absorption column. The end-life of DME was used as a substitute for diesel. Final direct CO_2_ emissions from the DME combustion were considered in order to compare it with diesel production impact. Other direct emissions consisted of CO_2_ in the flue gases from combustion of the purge gas. CO_2_ captured was stored, so it was not emitted.

## 3. Results and Discussion

### 3.1. Thermodynamic Assessment of Direct DME Synthesis from CO_2_-Rich Syngas

**Figure 4:**
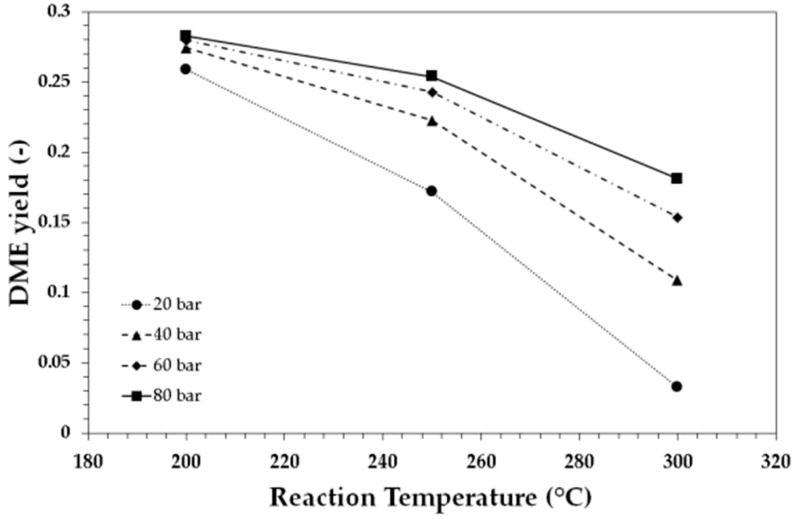
Theoretical equilibrium DME yield as a function of reaction temperature and pressure, for the raw syngas with the following molar composition: H_2_/CO = 0.87, CO_2_/CO = 0.94. Lines are only a guide for the reader.

In order to assess the effect of CO_2_ content, temperature and pressure on DME yield in the direct synthesis way, thermodynamics of the reaction were studied. The effects of reaction pressure and temperature on DME yield for the investigated raw syngas composition are reported in Figure 4.

The DME yield was calculated on carbon basis according to the following equation:

$$\mathrm{DME}\;\mathrm{yield}=\frac{\mathrm{moles}\;\mathrm{of}\;\mathrm{DME}\;\mathrm{produced}\;\times \;2}{\mathrm{moles}\;\mathrm{of}\;\mathrm{CO}\;\mathrm{and}\;{\mathrm{CO}}_{2}\;\mathrm{in}\;\mathrm{the}\;\mathrm{reactor}\;\mathrm{inlet}}$$

The trend clearly shows the negative effect of the temperature on DME yield. In fact, the direct DME synthesis is an exothermal reaction and is thermodynamically favored at low temperatures. For instance, for the investigated raw syngas at 40 bar, the theoretical equilibrium DME yield decreased from about 0.28 to about 0.11 by increasing the reaction temperature from 200 °C to 300 °C. Nevertheless, from a kinetic point of view, the reaction temperature was usually set at around 250 °C. Lower temperatures are not suitable for the conventional catalytic systems, while higher temperatures should also be avoided to retard catalyst deactivation by sintering [46]. On the contrary, higher pressures have a beneficial effect on DME yield. For instance, at 250 °C the theoretical equilibrium yield increased from about 0.17 to about 0.25 if the reaction pressure increased from 20 bar to 80 bar. The effect of pressure is of paramount importance since the reactant pressure is affected by the presence of inerts. In fact, the presence of a large amount of nitrogen (about 55%mol) in the investigated raw syngas stream had a negative effect on thermodynamics, as it lowered the partial pressure of the reactants and then reduced the theoretical equilibrium DME yield with respect to a syngas inert-free at the same total pressure.

**Figure 5:**
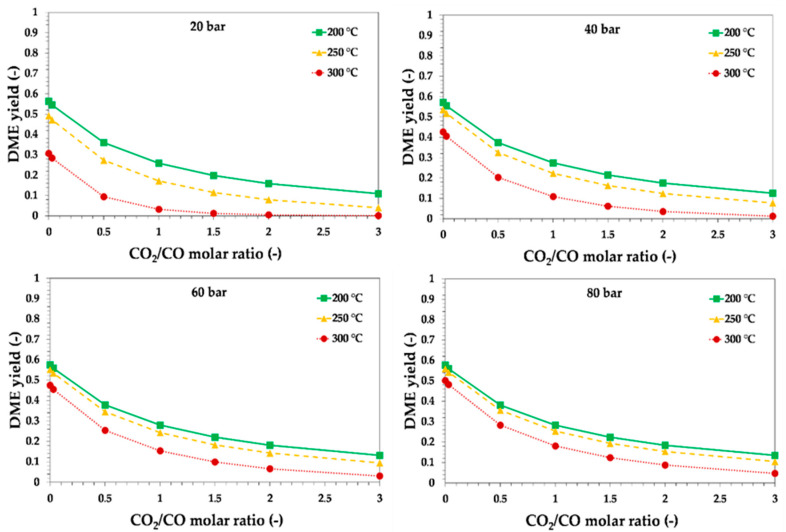
Theoretical equilibrium DME yield as a function of the initial CO_2_/CO molar ratio, reaction temperature and pressure, for syngas with H_2_/CO = 0.87. Lines are only a guide for the reader.

**Figure 6:**
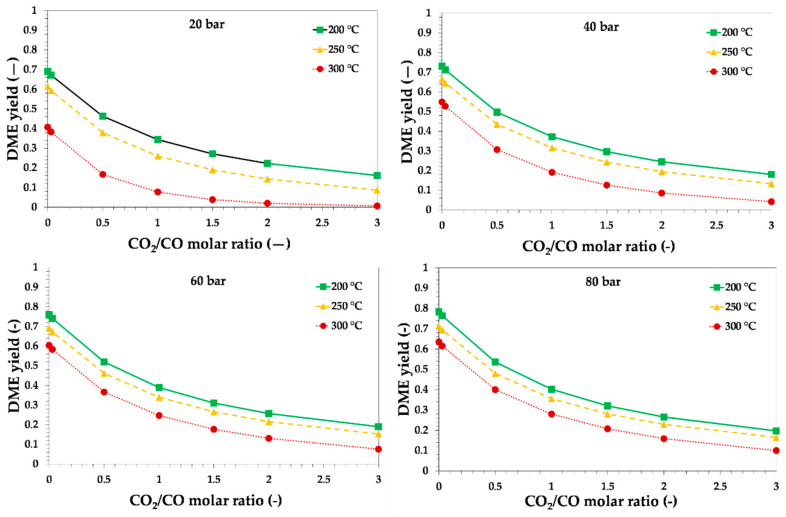
Theoretical equilibrium DME yield as a function of the initial CO_2_/CO molar ratio, reaction temperature and pressure, for syngas with H_2_/CO = 2. Lines are only a guide for the reader.

The presence of CO_2_ in the raw syngas also had a negative effect on thermodynamics, as the presence of CO_2_ promoted the reverse water–gas shift reaction, thus increasing the water production with a negative effect on thermodynamics of both methanol synthesis via CO_2_ hydrogenation and methanol dehydration to DME. Furthermore, the theoretical conversion of CO_2_ was lower than the theoretical conversion of CO, under the same reaction conditions, causing a lower DME yield. The CO_2_/CO ratio in the reactor inlet on theoretical equilibrium DME yield is reported in Figure 5 and Figure 6, for syngas with H_2_/CO ratios equal to 1 and 2, respectively.

The obtained results clearly indicate the negative effect of CO_2_ addition in terms of DME yield, especially at a higher temperature. In fact, the reverse water gas shift is an endothermic reaction and, hence, is more favored with the temperature increase. The negative effect of CO_2_ addition may be contrasted by a decrease in reaction temperature or an increase in reaction pressure. The adoption of lower reaction temperatures requires innovative catalytic systems with a higher activity than the conventional one. In this regard, the development of new catalysts for the production of DME from CO_2_-rich syngas or by hydrogenation of CO_2_ is receiving growing attention [47,48,49,50,51,52]. The research is mainly focused on the improvement of catalytic activity at low temperature and stability. As mentioned before, the presence of CO_2_ in the syngas increases the amount of water formed during the process, which has a negative effect on the resistance of the catalysts towards sintering.

Furthermore, the presence of CO_2_ increased the oxidation rate of the metal particles (e.g., copper), and a higher amount of hydrogen or more stable catalysts were then requested [53].

From a thermodynamic point of view, the increase in hydrogen content has a beneficial effect in terms of DME yield, as may be observed by comparing the calculated values reported in Figure 5 and Figure 6. A syngas with H_2_/CO ratio of 2 produced a DME yield higher than a syngas with H_2_/CO ratio of 1, under the same temperature, pressure and CO_2_ content.

**Figure 7:**
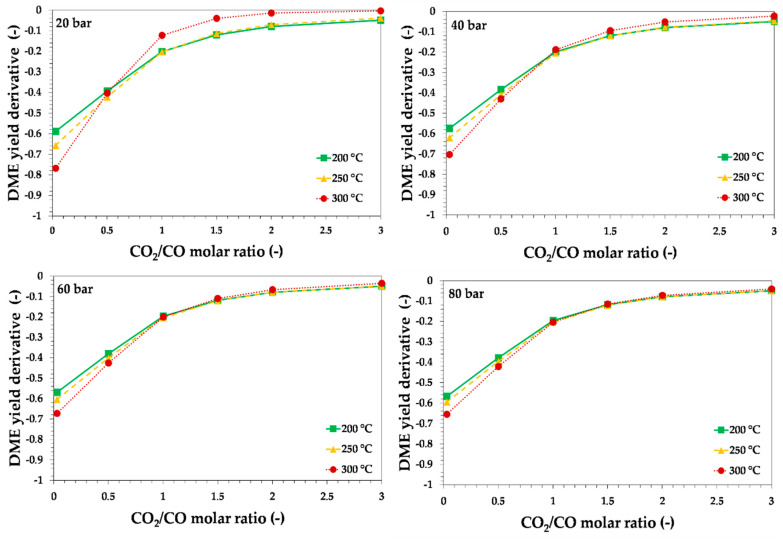
Theoretical equilibrium DME yield derivative with respect to CO_2_/CO molar ratio as a function of the initial CO_2_/CO molar ratio, reaction temperature and pressure, for syngas with H_2_/CO = 0.87. Lines are only a guide for the reader.

**Figure 8:**
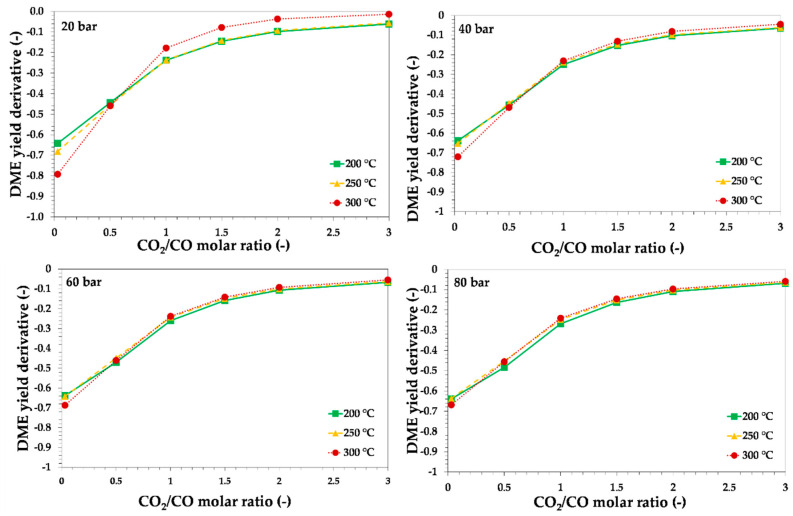
Theoretical equilibrium DME yield derivative with respect to CO_2_/CO molar ratio as a function of the initial CO_2_/CO molar ratio, reaction temperature and pressure, for syngas with H_2_/CO = 2. Lines are only a guide for the reader.

In order to better investigate the role of CO_2_ on thermodynamics, the derivative of DME yield with respect to CO_2_/CO ratio may be estimated, and results are reported in Figure 7 and Figure 8, for syngas with H_2_/CO equal to 1 or 2, respectively.

The results of the calculation indicate that the effect of increasing the CO_2_/CO molar ratio on DME yield was less evident for CO_2_/CO molar ratios higher than 2. Therefore, in other words, to increase the carbon dioxide recycling, if a syngas with CO_2_/CO = 2 is available, it may be convenient to add more CO_2_ with small differences in terms of DME yield decrease, from a thermodynamic point of view. The results in terms of DME yield also indicate that the reaction temperature’s negative effect on theoretical DME yield was less significant at a higher pressure and at higher H_2_/CO molar ratio.

### 3.2. Thermodynamic and Process Analysis of Direct DME Synthesis from CO_2_-Rich Syngas

After reaction thermodynamics analysis, the process simulation of the reactor with recycle was carried out. Considering WGS conversion, CO_2_ capture (post mixing with recycling) and the purge ratio changed in the ranges of Table 1.

The DME global yield was calculated on the total carbon atoms in the feedstock, according to the following equation:

$$\mathrm{DME}\;\mathrm{global}\;\mathrm{yield}=\frac{\mathrm{moles}\;\mathrm{of}\;\mathrm{DME}\;\mathrm{produced}\;\times \;2}{\mathrm{moles}\;\mathrm{of}\;\mathrm{CO}\;\mathrm{and}\;{\mathrm{CO}}_{2}\;\mathrm{in}\;\mathrm{the}\;\mathrm{clean}\;\mathrm{syngas}}$$

**Figure 9:**
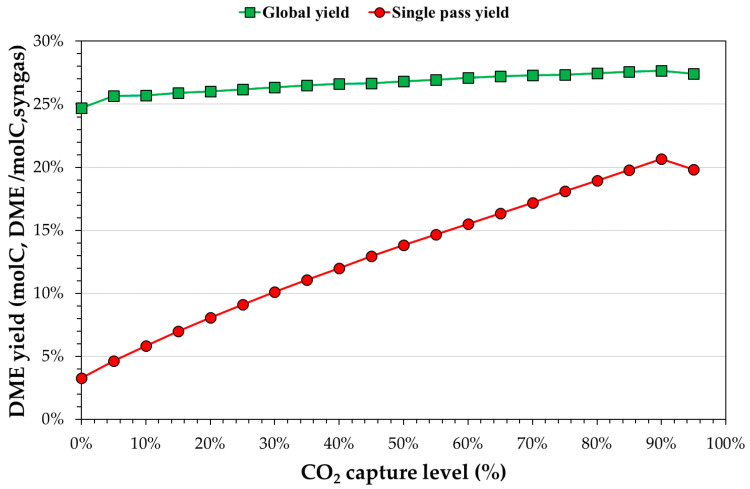
Theoretical equilibrium DME yield (global or single-pass) with respect to the CO_2_ capture, fixing the WGS conversion and the purge ratio to 0% and 10%, respectively.

**Figure 10:**
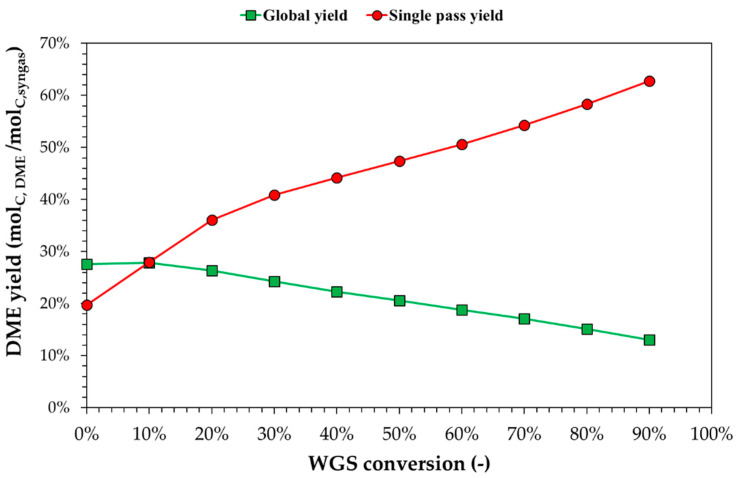
Theoretical equilibrium DME yield (global or single pass) with respect to the WGS conversion, fixing CO_2_ capture and the purge ratio to 85% and 10%, respectively.

**Figure 11:**
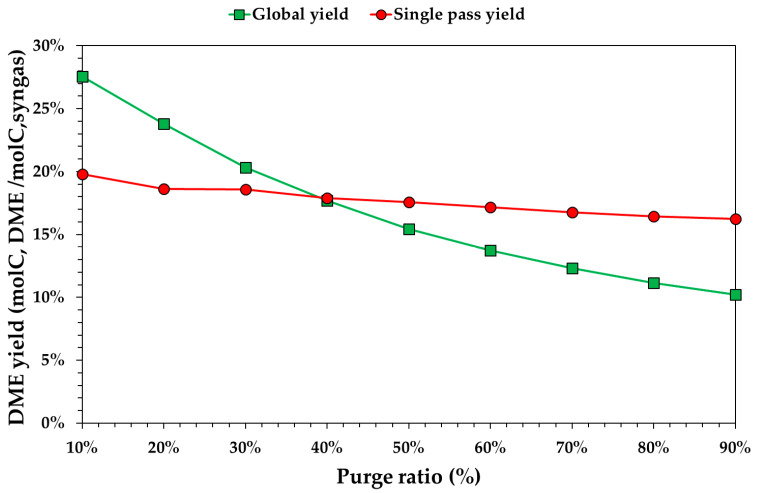
Theoretical equilibrium DME yield (global or single pass) with respect to the purge ratio, fixing WGS conversion and the CO_2_ capture to 0% and to 85%, respectively.

Figure 9, Figure 10 and Figure 11 show the sensitivity analysis results for different CO_2_ capture percentages, WGS conversion and purge ratios, respectively. The global and single-pass DME yields are shown. For each of these, the other two parameters were fixed equal to the best value. Figure 9 shows the yields varying the CO_2_ capture percentage. Increasing the CO_2_ captured increased the single-pass yield because of a better CO_2_/CO (lower) ratio. The highest value of single-pass yield was 90% of capture, for which the H_2_/CO ratio was the highest. For a higher CO_2_ capture percentage, the H_2_/CO ratio was lower (H_2_ is converted or lost in the purge), decreasing the single-pass yield.

The global yield increased from 24.7% (0% of capture) to 27.7% for a carbon capture of 85%. Increasing the CO_2_ capture increases the quantity of carbon (CO + CO_2_) available to convert to DME in the reactor decreases. This effect had a positive impact on the single-pass yield and, consequently, on the global yield, although the overall amount of (CO + CO_2_) that can be converted was lower.

Figure 10 shows yields varying by WGS conversion. In this case, the single-pass yield and global yield had two different behaviors. The single-pass yield increased the WGS conversion at higher H_2_/CO ratios, while CO_2_/CO increased slowly, thanks to CO_2_ capture. The global yield decreased, increasing the WGS conversion because CO_2_ removal is higher for high WGS conversion ratios. This subtraction of CO_2_ leads to the removal of “C” (CO + CO_2_) from the reaction system. For WGS conversion higher than 15%, the single-pass yield was higher than global yields due to the high CO_2_ capture.

Figure 11 shows yields varying by the purge ratio. In this case, both global and single-pass yields increased by increasing the purge ratio because the purge removed reactants. This effect was different on a single-pass and global yield. For a lower purge ratio the global yield was higher than the single-pass yield. For a purge ratio higher than 40%, the global yield was lower than the single-pass yield because CO and CO_2_ were lost in the purge. Single-pass yield decreased in a small range (from 19.8% to 16.2%) since the effect of the purge flowrate was not significant. This decrease was due to a greater impact on the CO_2_/CO ratio compared to the H_2_/CO ratio.

Optimal values for the CO_2_ capture percentage, WGS conversion and purge ratio were 85%, 0% and 10%, respectively. A carbon capture higher than 85% led to lower global yield, and further carbon capture is not easy at low partial pressure. The optimal WGS conversion was equal to 0% in order not to convert CO to CO_2_, which is removed after recycle mixing. The best purge ratio was the lowest, considering it was not possible to prevent an accumulation of inerts in the purge, and a feasible value of 10% was chosen.

### 3.3. Process Simulation of DME Synthesis from Digestate-Derived Syngas

**Figure 12:**
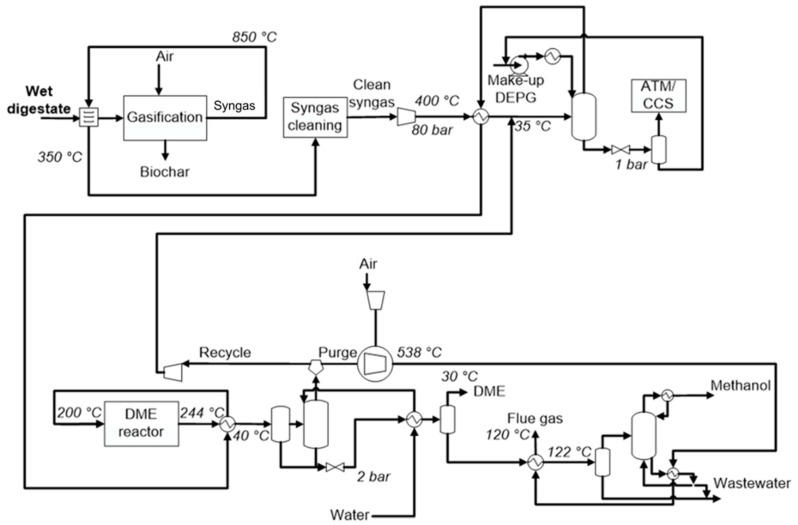
Flowsheet diagram for the optimal process result.

In the process simulation of DME synthesis from digestate-derived syngas, optimal process parameters were set. Figure 12 shows the final flowsheet was without the WGS step, and with a carbon capture equal to 85%, the purge ratio was fixed to 10%. In the reported flowsheet, a heat-exchanger unit was also proposed in the case of wet digestate. As mentioned above, the syngas adopted for simulation was produced from air gasification in an autothermal gasifier using a digestate with a 10%wt of moisture as feedstock. In the case of higher moisture content, the sensible heat of the produced syngas may be used to reduce the moisture content at the desired value, when possible. The syngas temperature was assumed to decrease at a value higher than 350 °C, in order to prevent tar condensation. The effect of digestate moisture on carbon dioxide emission is discussed later.

Table 3 shows the main process parameters as results of the detailed process simulation. The equilibrium temperature was 244 °C due to the high molar fraction of inerts. Pressure drop in the reactor was supposed equal to 2 bar, so the outlet pressure was 78 bar. This pressure was used also in the absorption column, and it is the inlet pressure to the recycle compressor. DEPG solvent flowrate was equal to 9.9 t/h, and process water for the absorption column was 1.35 t/h. Carbon dioxide captured by Selexol^®^ process was 11.1 t/h. This value represents 60% of the total CO + CO_2_ of the fresh syngas. Considering CO_2_ was 48% of the total CO + CO_2_ in the fresh syngas, the DME synthesis transformed CO to CO_2_ to add other 12% of “C” to carbon capture. The molar ratio between recycled syngas and fresh syngas was equal to 5.8, derived from the fixed purge ratio (10%).

The global yield to methanol was negligible. Mass yield to DME was equal to 7.5%wt of the digestate feedstock. In the purge stream, 12% of CO + CO_2_ was from the fresh syngas (20% of this was CO_2_).

Because of the low purge ratio used (10%), the ratio between recycled syngas and the fresh syngas was very high (5.8). This can be due to the very high synthesis reactor volumes (and catalysts mass) required.

The low purge ratio also had an effect on electricity production. For this case, the total energy power produced was not sufficient to cover compressor energy requirements. Produced energy can cover 87% of the total electricity required. A difference of 73 kWe between consumed and produced energy was found.

### 3.4. Environmental Assessment

**Figure 13:**
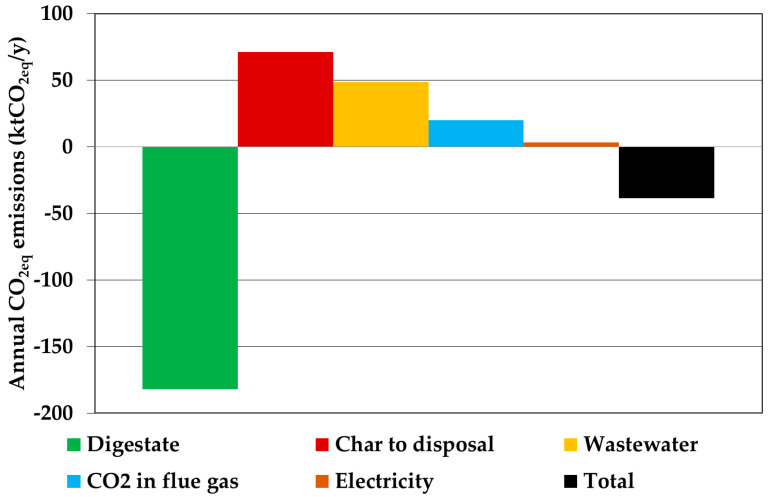
Equivalent CO_2_ emissions of the optimal flowsheet.

For the environmental assessment, the CO_2_ equivalent emissions were used to estimate the impact of both direct and indirect emission and savings. Figure 13 shows the annual CO_2eq_ emissions related to the main process streams. Savings from digestate utilization were higher than emissions from char disposal because about 61% of the digestate mass was converted to syngas. The wastewater was the second main emission with about 49 ktCO_2_/yr. The combustion of purge gas had direct emissions of 20 kt/y of CO_2_, which was lower than the captured amount of CO_2_ (31 kt/y). Because of the low purge ratio value (10%), this emission was low, but the produced electricity in the plant was not sufficient to cover consumption of the compressors and other energy-intensive equipment. For this reason, about 3 kt/y of equivalent CO_2_ was emitted when fossil-source was used for electricity generation.

The total final emissions were negative (−39 ktCO_2eq_/y) mainly thanks to the high savings from digestate utilization (−182 ktCO_2eq_/y).

**Figure 14:**
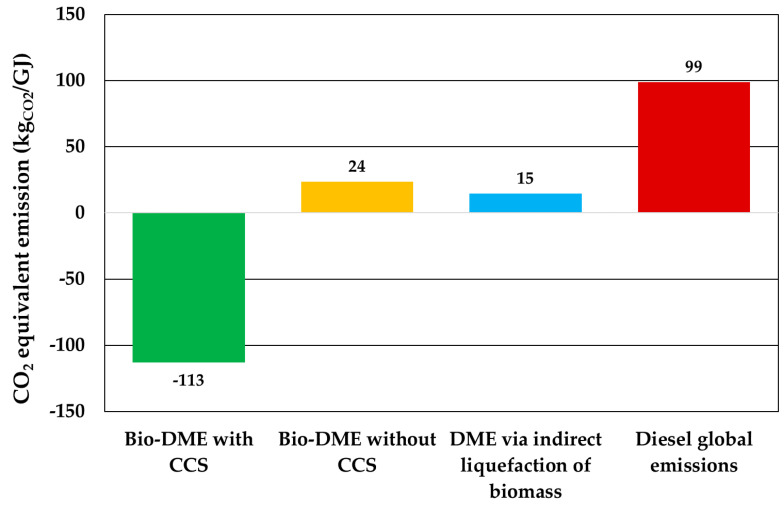
Environmental impact comparison between bio-DME from digestate with CCS, bio-DME from digestate without CCS, DME from biomass indirect liquefaction [56] and diesel.

Figure 14 shows the comparison between four equivalent fuel sources in terms of emissions per GJ of energy. The worst case was the equivalent fossil-based fuel (diesel) with 99 kgCO_2eq_/GJ, considering the CO_2_ emission after combustion [54]. Tan et al. evaluated the impact of the DME production using the indirect liquefaction of biomass based on the full life-cycle (including biogenic CO_2_ and end-use emissions). In the work, a global emission of 15 kgCO_2eq_/GJ was assessed. This value is higher than the environmental impact found in the present work (−113 kgCO_2_/GJ), considering the end-use emissions. A further case was investigated for the comparison of Figure 13, and it consists of evaluating the impact of DME from digestate syngas without CCS, emitting the CO_2_ separated before the synthesis reactor. For this case, the electricity consumption was lower (5.2 MWe), but the direct CO_2_ emissions were higher (20 kt/y from flue gases and 31 kt/y from the carbon capture unit). Globally, an emission of 24 kgCO_2eq_/GJ was found, higher than those in the base case (with CCS) and Tan et al.’s work.

Another recent work to make the comparison is Uddin et al. [55]. This study described the techno-environmental analysis of dimethyl ether (DME) production via methanol dehydration where methanol was synthesized through bi-reforming of CH_4_ from landfill gas. Results showed a negative value of –32 kgCO_2eq_/GJ from cradle to gate and a positive value of 35 kgCO_2eq_/GJ from cradle to grave. This last value is higher than both cases in the present work, with or without the carbon dioxide storage section. Without considering the end-use emissions (cradle-to-gate), lower values of impact were obtained: −179 and −43 kgCO_2eq_/GJ with or without CCS, respectively.

**Figure 15:**
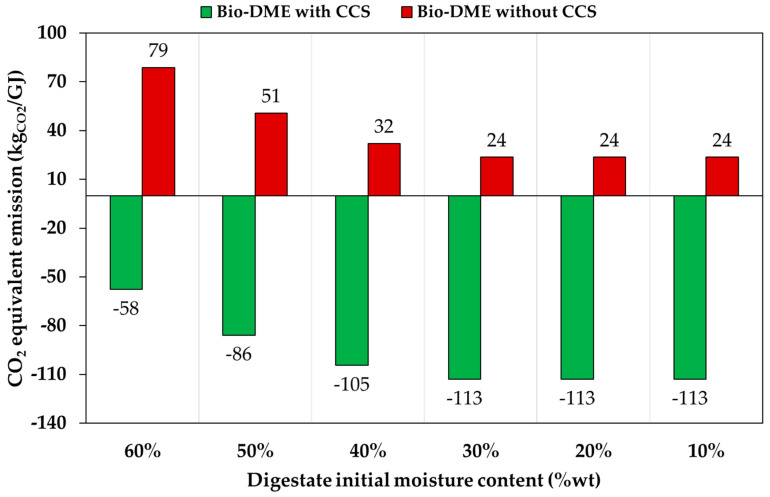
Effect of initial digestate moisture content on carbon dioxide emission of the proposed process plant for DME synthesis.

As previously mentioned, the above calculation was carried out by considering a digestate with a moisture content equal to about 10%wt. It is well known that water content may be also as high as 60% [57]. In that case, a drying unit is requested in order to reduce the moisture content to a desired value, e.g., 10%wt. As described above, the sensible heat of the produced syngas may be used, and if this is not sufficient, additional heat may be produced by natural gas or bio-methane combustion, with an increase in carbon dioxide emissions. In order to take into account this aspect, the carbon dioxide emissions were re-calculated as a function of digestate initial moisture, and results are reported in Figure 15. Results clearly indicate that a negative value of carbon dioxide emissions was obtained for any moisture content value in the case of CCS-based plants. On the contrary, the carbon dioxide emissions strongly increased for initial moisture contents higher than 30%wt. In fact, for moisture contents up 30%wt, the sensible heat of produced syngas was sufficient for the drying step, while combustion of natural gas or bio-methane was requested for higher water contents. Of course, carbon dioxide emissions related to digestate drying may be avoided by using renewable-fueled technologies, e.g., concentrating solar power devices [58].

## 4. Conclusions

In this work, the environmental assessment of dimethyl ether (DME) production using digestate as raw material for the gasification process was carried out. The organic fraction of municipal solid waste can be used in the anaerobic digesters to obtain biogas/biomethane, and the solid waste fraction (digestate) can be gasified to obtain syngas. The valorization of the syngas can happen only with specific H_2_/CO/CO_2_ ratios, in order to optimize the next product synthesis.

An original sequence of steps was applied in this work to obtain the optimal process configuration. First a thermodynamics analysis on a single-pass DME direct synthesis reactor was carried out. The synthesis reactor with recycle was evaluated on the basis of specific composition of the digestate-based syngas, and cases considering the WGS and carbon capture steps were also assessed. In the next step, the global process simulation with optimal process parameters found in the previous stages was assessed, evaluating all input and output mass and energy flows. Finally, the environmental impact analysis was applied using mass and energy balances of the process simulation and the CO_2_ equivalent emissions relationships.

From the thermodynamics analysis, higher H_2_/CO molar ratios improve DME equilibrium yield. Furthermore, high CO_2_ content reduce DME equilibrium yield, although the effect of the increase of the CO_2_ amount is less significant for high CO_2_/CO molar ratios. Temperature and pressure optimal ranges were 200–250 °C and 60–80 bar, respectively. Adding the recycle and possibility of having the WGS stage and the carbon capture by Selexol^®^, an optimal WGS conversion and carbon capture equal to 0% and 85% were found, respectively. The final simulation showed the electricity production was insufficient to cover the energy required by compressors (until 80 bar), and the yield to DME was equal to 0.075 kg_DME_/kg_DIGESTATE_. In terms of environmental impact, considering the carbon capture and storage (CCS) of CO_2_ a value of −113 kgCO_2_/GJ was found. This negative value derived from high CO_2_ savings from digestate utilization, instead of disposal in a landfill. From the comparison of the impact with similar bibliography cases, digestate is the optimal way to produce DME if the CCS is carried out. Finally, digestate is a good feedstock to obtain an environmental benefit, but the yields to products have to be improved to make the digestate valorization a commercial alternative to the fossil sources. Furthermore, the initial moisture content in the digestate may have an impact on carbon dioxide savings, which are limited by using renewable-fueled drying technologies.

## Figures and Tables

**Table 1:** Process parameter values.

Process Parameter	Units	Value/Range		Reference
Syngas composition	%	CO_2_	12.9	[15]
		C_2_H_6_	1.3	
		H_2_	11.9	
		CH_4_	4.5	
		CO	13.7	
		N_2_	55.7	
Syngas flowrate	t/h	16.3		[15]
WGS conversion	%	0–95		-
CO_2_ capture	%	0–95		-
Purge split ratio	%	10–90		-
HT-WGS temperature	°C	400		[35]
LT-WGS temperature	°C	200		[35]
DME synthesis equilibrium temperature	°C	200–300		[22]
DME synthesis pressure	bar	20–80		[32]
Digestate flowrate	t/y	100,000		[35]
DME purification columns pressure	bar	2		[36]
Selexol^®^ separation temperature	°C	35		[37]
H_2_O/DME absorption column ratio	mol/mol	33		[36]

**Table 2:** Equivalent carbon dioxide emission of the main process streams.

Process Stream	Units	Value	Reference
Electricity required	kgCO_2eq_/MWhe	600	[45]
MeOH	kgCO_2eq_/t	−1643	[45]
Digestate	kgCO_2eq_/t	−1821	[44]
Wastewater	kgCO_2eq_/t	500	[45]
Solid gasification residues	kgCO_2eq_/t	1821	[44]
Process water	kgCO_2eq_/t	6.5	[35]

**Table 3:** Process results.

Process Parameter	Units	Value
WGS conversion	%	0
CO_2_ capture	%	85
Purge split ratio	%	10
DME reactor outlet temperature	°C	244
DME reactor outlet pressure	bar	78
Pure DME flowrate	t/y	7496
Pure methanol flowrate	t/y	82
Wastewater	t/y	97,153
Process water	t/y	97,200
CO_2_ captured and stored	t/y	30,923
Solid residues to disposal	t/y	39,000
DEPG flowrate	t/h	9.9
Flue gases	t/h	36
Recycle/fresh syngas stream	mol/mol	5.8
Energy power produced	MWe	4.8
Energy power consumed	MWe	5.6
